# Associations between alcohol consumption and spending on gambling like mechanisms in video games

**DOI:** 10.1038/s41598-025-15301-4

**Published:** 2025-08-31

**Authors:** Lucy C. East, James D. Sauer, Emily Lowe-Calverley, Lauren C. Hall, Benjamin J. Martin, Aaron Drummond

**Affiliations:** School of Psychological Sciences, University of Tasmania, Churchill Avenue, Sandy Bay, Hobart, TAS Australia

**Keywords:** Video gaming, Loot boxes, Gambling, Problem gambling, Internet gaming disorder, Psychology, Human behaviour

## Abstract

Loot boxes are purchasable digital containers in video games that hold randomised rewards. Many loot boxes meet both psychological and legal criteria for gambling. Previous studies have linked risky engagement with, and increased spending on, loot boxes with both problem gambling and excessive gaming symptomatology. Given similarities between loot boxes and conventional gambling, and the well documented relationship between alcohol and gambling, this study examined whether loot box spending was associated with drinking behaviours. In a pre-registered study, participants (*N* = 199) from Australia, Aotearoa (New Zealand), and the United States completed an online survey assessing alcohol consumption, loot box spending behaviours, and problem gambling and gaming symptomatology. Small-to-moderate positive correlations between drinking during gaming sessions and increased risky loot box engagement and spending were observed. In contrast, measures of problematic alcohol consumption did not correlate with increased spending or risky engagement with loot boxes. Results suggest that drinking alcohol while gaming may be associated with risky engagement with, and increased spending on, loot boxes.

## Introduction

Video games are a popular pastime, with approximately two-thirds to four-fifths of individuals regularly playing video games across Australia and the United States (US)^1,2^. Recently, concern has grown amongst parents, policy makers, and researchers about the inclusion of gambling-like mechanisms within video games^3^. Loot boxes are randomised digital containers that hold one or more random rewards^3^and many loot boxes are purchasable for real world money. The psychological mechanisms underpinning loot boxes are strikingly similar to those underpinning conventional gambling, such as the use of powerful intermittent reinforcement schedules to drive the chance-based distribution of unknown prizes in exchange for a cash consideration^3,4^. Research to date has observed robust associations between addictive behaviours (including problem gambling and excessive gaming) and loot box expenditure^5^. Given that loot box purchasing is partially associated with impulsive behaviours^6^and that behavioural addictions often share comorbidities, this study sought to determine if the relationship between excessive gaming and gambling could extend to other potentially addictive behaviours such as alcohol consumption.

## Associations between loot box spending and gambling

The variable-ratio of reinforcement used in loot boxes is the same reinforcement schedule underpinning slot machines and lotteries^3,6^and has long been known to result in the rapid acquisition and frequent repetition of behaviours in pursuit of rewards, and to produce behaviours that are highly resistant to extinction^7^. Loot box rewards often allow players to enhance game play through visual alterations to the game (e.g., through character customisation), or gaining competitive advantage (e.g., via more powerful tools and weapons)^3^. Often, the most desirable or powerful rewards are also deliberately manipulated to be the rarest^4^. Therefore, more useful or rarer items become particularly desirable to possess^3^. Like in conventional gambling, this is thought to foster ‘chasing behaviour’ where a player may continue to buy loot boxes rapidly, mistakenly believing they are more likely to receive a desirable reward on the next purchase^8^. However, just as in conventional gambling, there is no cumulative likelihood of a player receiving a rarer in-game item, and the likelihood of receiving a high-value item can be lower than 1%^4^.

Furthermore, loot boxes have been found to meet both the psychological and legal criteria for gambling^4^. Using Griffiths’s (1995) criteria for distinguishing gambling from other forms of risk-taking behaviour^9,10^. Drummond and Sauer (2018) found that, for almost half of the mainstream games containing loot boxes released in 2016–2017, those loot boxes met all the psychological criteria to be considered a form of gambling, and approximately a quarter allowed players to cash out winnings for real world currency, often a requirement for legal definitions of gambling^3^. In a follow-up study, Drummond et al. (2020) established that many loot box systems meet the legal definitions of gambling, that is price, consideration, and chance.

Concordantly, loot box spending is associated with other addictive behaviours, including both problem gambling symptomatology and symptoms of excessive gaming^6,8,11,12^; results which are confirmed to represent robust small-to-moderate associations under meta-analysis^5^. Although causal direction remains uncertain (i.e., whether loot boxes cause migration to gambling or are just disproportionately purchased by individuals at increased risk of gambling harms), both directions could be problematic, representing either the potential for migration into conventional gambling, or the disproportionate purchasing of loot boxes by a vulnerable group. Indeed, recent findings show that those at greatest risk of gambling harm spend the most on loot boxes irrespective of their income band^13,14^. Furthermore, recent evidence suggests that spending on loot boxes appears to be associated with greater migration into conventional gambling six months later^15,16^.

The association between loot box spending and problem gambling symptomatology has been replicated in Australian, Aotearoa (New Zealand), and US samples with results again indicating small-to-moderate positive associations between problem gambling symptomatology, risky interactions with loot boxes, and increased spending on loot boxes^11^. Additionally, there was a significant interaction between excessive gaming and problem gambling symptomatology and loot box spending^11^. Players with high levels of both excessive gaming and problem gambling symptoms spent more on average on loot boxes than individuals with low scores on one or both of these measures^11^. The present project seeks to extend this research by further clarifying the relationship between loot box spending and other addictive behaviours.

## Relationship between alcohol consumption and gambling

The consistently observed associations between the addictive behaviours of gambling and excessive gaming, and loot box expenditure and risk-taking, raise the question as to whether associations could extend to other potentially addictive behaviours, such as alcohol consumption. Alcohol and gambling frequently co-occur^17^. Impulsive responses (a tendency to act on unplanned actions) can become more prevalent when alcohol is consumed, due to a supressing effect on the inhibitory control centres of the brain^18^. Impulsivity is a key component in developing and maintaining addictive behaviours such as problem gambling, therefore alcohol intoxication may plausibly exacerbate problematic gambling behaviours^18^. Alcohol consumption is also positively associated with increased size of gambling bets and more rapid depletion of funds^19^.

However, risk-taking behaviours are unlikely to be caused by alcohol-induced impulsivity alone^22^. Research to date indicates that there appears to be a substantial overlap between an individuals’ neurobiological makeup and impulsive behaviour, suggesting that trait-impulsivity (a personality trait that results in a tendency towards unplanned actions), may dispose individuals towards risk-taking regardless of alcohol consumption^18,20,21^. A recent systematic review and meta-analysis found no significant effect of acute alcohol consumption on risk-taking behaviour whilst gambling^22^. This suggests that both biological and psychological mechanisms likely underpin the relationship between gambling and alcohol consumption, and that both a neurological or cognitive predisposition to gambling and acute alcohol intoxication may be needed to increase the likelihood of risk-taking behaviour^22^.

In sum, there is a substantial body of evidence to suggest a relationship between alcohol consumption and conventional gambling, such that drinking while gambling has been linked to overspending and increased impulsive behaviour while gambling^19^. In turn, there is a reliable meta-analytic association between problem gambling symptoms and spending on loot boxes^5^. Moreover, while unidimensional constructs of impulsivity do not appear to be associated with loot box spending^23,24^some aspects of impulsivity are associated with increased loot box spending^14^. Given the psychological similarities between loot boxes and conventional gambling, it is important to determine whether this relationship between alcohol and conventional gambling extends to loot box spending. To this end, we examined the association between both habitual and acute alcohol consumption and loot box spending.

### Present study

The present study aimed to determine whether alcohol consumption is associated with in-game spending on loot boxes. In accordance with the literature showing clear associations between loot box spending and problem gambling, and between gambling and alcohol consumption, we predicted that the amount of money spent on loot boxes in the past month would:

1. Correlate positively with the amount of alcohol consumed (i.e., habitual alcohol consumption).
2. Correlate positively with the amount of alcohol consumed during gaming sessions (i.e., acute alcohol consumption).

We also predicted that risky engagement with loot box spending would:

3. Correlate positively with the amount of alcohol consumed (i.e., habitual alcohol consumption).
4. Correlate positively with the amount of alcohol consumed during gaming sessions (i.e., acute alcohol consumption).

Furthermore, we predicted that:

5. Correlate positively with the amount of alcohol consumed (i.e., habitual alcohol consumption). The amount of alcohol consumption and problem gambling symptomatology would interact, such that players with higher alcohol consumption (+ 1*SD*) and higher problem gambling symptoms (+ 1*SD*) will spend more on loot boxes than those low in one or both of these variables.
6. The amount of alcohol consumption and excessive gaming symptomatology would interact such that players with higher alcohol consumption (+ 1*SD*) and higher problem gaming symptoms (+ 1*SD*) will spend more on loot boxes than those low in one or both of these variables.

## Methods

### Preregistration

This study’s hypotheses, data collection, and analysis plan were preregistered and can be accessed via the Open Science Framework (https://osf.io/8adbs).

### Participants

Two hundred participants who were over 18 years of age residing in Australia, Aotearoa (NZ), or the US were recruited via Prolific Academic. To ensure the results remained generalisable to the broader video gaming population, inclusion criteria required participants to be: (1) 18 years of age or older, (2) self-identified as active video gamers, and (3) residents of one of the following countries: the United States of America, Australia, or Aotearoa (NZ). We also excluded participants who answered “true” to a mischievous responding question “I once owned a three-headed dog.” No additional exclusion criteria were applied. Ninety-seven (97) participants were from Australia, 26 participants were from Aotearoa, and 77 participants were from the United States. One participant was removed due to failing the attention check question (*N* = 199). Average age of participants was 38 years (*SD* = 12) with 56.3% identifying as male (*n* = 112), 42.2% identifying as female (*n* = 84), and 1.5% of participants identifying as non-binary or other (*n* = 3). These demographics align with estimates of the average age of video gamers being between 34 and 40 years old in the general population^1,2,11^and are consistent with samples used in previous work on loot box spending^3^.

### Ethics

Ethical approval was reviewed and granted by University of Tasmania Human Research Ethics Committee (HREC), approval number H0029174. All research was conducted in accordance with the relevant guidelines and regulations and informed consent was obtained from each participant prior to participation.

### Design and analysis

Cross-sectional, correlational data were collected as part of an online questionnaire via Prolific. Participants retained anonymity and were asked to provide only age, gender, and country of residence. All measures were continuous and were analysed using Bayesian correlations and regressions. This study jointly collected data for another study supervised by the last author. Only specified variables within the preregistration document were analysed and are relevant to the present project, and we provide a complete account of all analyses undertaken for this project.

### Procedure

After providing informed consent, participants specified the amount they had spent in the previous month in their national currency on loot boxes, the amount of alcohol they consumed during their most recent gaming session, and estimated the percentage of time that they consumed alcohol during gaming sessions. Following this, participants all completed the Risky Loot Box Index (RLI)^6^the Problem Gambling Severity Index (PGSI)^25^the Alcohol Use Disorders Identification Test (AUDIT)^26^and the Internet Gaming Disorder Checklist (IGD)^11^ in this order prior to receiving debriefing.

### A-priori power analysis

An a-priori G*Power analysis determined that to detect correlations of *r* = .2, with a power of 80% at alpha level 0.05, a minimum sample size of 150 people was necessary. A sample size of 200 was selected to ensure this number was exceeded after exclusions were applied.

### Measures

Participants were asked to respond to an online questionnaire, including validated measures and researcher-initiated questions. Questions tapped into key constructs of drinking behaviour, problem gambling symptoms, and loot box spending. We also included measures of video game spending more generally for exploratory purposes, but as we followed the preregistration plan, these variables were not used in this study.

### Measures of loot box spending

Participants were asked to specify the amount (dollars and cents) they have spent on loot boxes in the past month in their own currency (e.g., “In the past month, how much money have you spent on loot boxes? ). On 15/8/2023 all responses from Australia and Aotearoa (NZ) were converted into $USD. Currency conversion rates on this day were USD = 0.65*AUD and USD = 0.60*NZD respectively.

### Risky loot box index

The RLI is a validated and standardised 5-item questionnaire used to measure a person’s risky engagement with loot boxes in video games^6^. Participants were asked to rate their responses to statements (e.g., “I frequently play games longer than I intend to, so I can earn loot boxes”) on a 5-point Likert scale from strongly disagree (1) to strongly agree (5)^6^. Participants received a score out of 25, with higher scores indicating riskier engagement with loot boxes^6^.

### Problem gambling severity index

The PGSI is a validated and standardised measure of risky behaviour in problem gambling^25^. The PGSI consists of 9 questions (e.g. “Have you ever bet more than you could afford to lose?”) rated on a 4-point Likert scale from never at all (0) to almost always (3)^25^. Participants received a score out of 27 with higher scores indicating greater symptoms of problematic gambling behaviour^25^.

### Problematic alcohol consumption

The AUDIT is a validated and standardised measure indexing consumption of alcohol, drinking behaviours, and alcohol related problems^26^. The AUDIT consists of 10 questions (e.g., “How often have you had six or more standard drinks on one occasion?”) rated on a 5-point Likert scale from never (0) to daily or almost daily (4)^26^. Participants received a score out of 40 with higher scores indicating greater alcohol use^26^.

### Measures of session drinking

Participants were asked to estimate “how frequently do you consume alcohol whilst gaming?” (e.g., percentage of time spent consuming alcohol whilst gaming) rated from 0 to 100% in 10% intervals, as well as “how many standard drinks of alcohol did you consume during your last gaming session?” specified in number of standard drinks.

### Excessive gaming symptomatology

The IGD is a validated and standardised measure of excessive gaming behaviours^11^. The IGD consists of 9 items (e.g., “I feel irritable, anxious or sad when I am unable to game” rated on a 4-point Likert scale from not true at all (0) to very true (3))^11^. Participants received a score out of 27 points with higher scores indicating greater video game engagement^11^.

## Results

All data are openly available on the OSF (https://osf.io/fwp4t/files/osfstorage/6758d7742158a00950f73a53). All hypotheses were tested using Bayesian analyses, which allow evidence to be evaluated on a continuous scale using Bayes Factors (BF). The BF quantifies the strength of evidence in favour of the alternative or null hypothesis, with, BF_10_ indicating evidence in favour of the alternate hypothesis^27^. For correlations, Pearson’s *r* was used as the index of effect size. As all correlational hypotheses were directional, and variables were predicted to correlate positively, wherever possible, BF_+0_ was used, which indicates evidence in favour of a positive correlation. Evidence in favour of the alternative (or null) can be categorised as follows; BF 1/3–1 = anecdotal evidence for H_0_, BF 1 = no evidence favouring the null or alternative, BF 1–3 = anecdotal evidence for H_1_, BF 3–10 = moderate evidence for H_1_, BF 10–30 = strong evidence for H_1_, BF 30–100 = very strong evidence for H_1_, and BF > 100 = extreme evidence for H_1_^28^. In this sample, high levels of skew and kurtosis were observed in the following variables: number of standard drinks, frequency of drinking while gaming, loot box spending, AUDIT, and PGSI scores (see Table 1). As preregistered, variables violating normality assumptions (skewness or kurtosis +/- > 2.0) were rank ordered prior to analysis, and Spearman’s correlation was conducted to reduce Type I error risk^29,30^. Spearman’s rho (*r*_*s*_) is reported when used; otherwise, Pearson’s *r* is reported.

All analyses were conducted using Jamovi^31^.

**Table 1:** Descriptives for all variables.

Variables	*n*	*M*	*SD*	Min	Max	Skew	Kurtosis
Age	188	37.94	11.65	20	72	0.61	-0.16
No. standard drinks	197	0.88	1.61	0	12	3.22*	14.71*
Frequency of drinking while gaming	195	10.82	18.76	0	100	2.4*	5.67*
AUDIT	192	4.92	5.53	0	35	2.27*	6.78*
PGSI	195	2.21	3.78	0	24	2.43*	7.14*
IGD	198	5.52	5.14	0	22	0.83	-0.11
Loot box spending ($USD)	199	10.08	26.12	0	200	4.81*	28.91*
RLI	196	9.07	4.97	5	25	1.18	0.51

*M* = mean, *SD* = standard deviation, Min = minimum, Max = maximum, * = skew or kurtosis exceeding 2.0, no. standard drinks = number of standard drinks consumed during previous gaming session, frequency of drinking while gaming = estimation of the percentage of the time participants are engaging in gaming and alcohol consumption concurrently,AUDIT = Alcohol Use Disorder Identification Test,PGSI = Problem Gambling Severity Index,IGD = Internet Gaming Disorder Checklist, Loot box spending ($USD) = amount spent on loot boxes in United States dollar amount during the past month, and RLI = Risky Loot Box Index. ^a^ Due to missing data on some variables, the number of responses to each variable varies slightly.

Bayesian correlations assessed the relationships between alcohol consumption across the domains of AUDIT scores, number of standard drinks and frequency of drinking while gaming on the dependent variables, risky engagement with loot boxes (RLI score) and loot box spending (see Table 2). Contrary to H1, there was strong evidence against a positive association between AUDIT scores and loot box spending (*r*_*s*_ = − 0.01, BF_+ 0_ = 0.09). In support of H2, there was moderate evidence for a weak positive association between frequency of drinking and loot box spending (*r*_*s*_ = 0.17, BF_+ 0_ = 3.11). There was also anecdotal evidence to suggest a weak positive association between the number of standard drinks consumed whilst gaming and increased loot box spending (*r*_*s*_ =0.16, BF_+ 0_= 1.88). Anecdotal evidence was also observed in favour of H3, suggesting a small positive correlation between AUDIT scores and risky engagement with loot boxes (*r*_*s*_ = 0.15, BF_+ 0_= 1.31).

**Table 2:** Results of bayesian correlations comparing AUDIT scores, frequency of drinking, no. standard drinks, PGSI and IGD scores to loot box spending and risky loot box index scores.

Variables				Loot box spending ($USD)	RLI score	
AUDIT scores		*r*_*s*_		− 0.01	0.15	
		BF_+ 0_		0.09^a^	1.31	
		95% CI		[0.00, 0.16]	[0.28, 1.31]	
Frequency of drinking		*r*_*s*_		0.17	0.3	
		BF_+ 0_		3.11	1302.06	
		95% CI		[0.3, 0.04]	[0.42, 0.16]	
No. standard drinks		*r*_*s*_		0.16	0.27	
		BF_+ 0_		1.88	246.63	
		95% CI		[0.29, 0.03]	[0.39, 0.13]	
PGSI scores		*r*_*s*_		0.22	0.35	
		BF_10_		10.93	18,364.78	
		95% CI		[0.35, 0.08]	[0.47, 0.22]	
IGD scores		*r*_*s*_		0.41	0.46	
		BF_10_		4,160,401.76	539,377,539.41	
		95% CI		[0.52, 0.28]	[0.56, 0.34]	

All data are rank ordered, except RLI and IGD scores. This renders all correlations as Spearman correlations except for the association between IGD and RLI scores which is a Pearson’s correlation. 95% CI = Credible intervals, loot box spending ($USD) = amount spent on loot boxes in United States dollar amount during the past month, RLI = Risky Loot Box Index, AUDIT = Alcohol Use Disorder Identification Test, frequency of drinking = estimation of what percentage of the time participants are engaging in gaming and alcohol consumption concurrently, no. standard drinks = number of standard drinks consumed during last gaming session, PGSI = Problem Gambling Severity Index, and IGD = Internet Gaming Disorder Checklist, *r*_*s*_ = Spearman’s rho. ^a^ Indicates evidence in favour of the null hypothesis.

In support of H4, there was extreme evidence for a small-to-moderate positive correlation between the number of standard drinks consumed during gaming sessions and increased risky interactions with loot boxes, *r*_*s*_ = 0.27, BF_+ 0_ = 246.63. Similar results were found in terms of frequency of drinking whilst gaming and risky engagement with loot boxes (see Table 2). Of the sample, 82.91% of our participants indicated that they had consumed alcohol, and 44.22% of participants indicated they had consumed alcohol whilst playing video games. Notably, only 18.59% of participants reported engaging both target behaviours, that is consuming alcohol while gaming and interacting with loot boxes. Taken together, these results suggest that acute alcohol consumption during play sessions (but not habitual drinking behaviour) is associated with increased spending on loot boxes. A Bayesian linear regression tested whether indexed scores of alcohol consumption (AUDIT) and problem gambling symptomatology predicted loot box spending, and indicated that, counter to predictions (H5), the model of best fit included only problem gambling symptomatology. When Bayes factors were compared as the ratio of PGSI scores over the interaction term, strong evidence was found favouring the model that included only problem gambling symptomatology, over the model including both main effects and the interaction term, for predicting loot box spending (BF_10_ = 12.16).

A Bayesian linear regression tested whether indexed scores of alcohol consumption (AUDIT) and excessive gaming symptomatology (IGD) predicted loot box spending and, contrary to H6, indicated that the model of best fit included only excessive gaming symptomatology. Again, strong evidence was found favouring the model including only excessive gaming symptomatology, over the model including both main effects and the interaction term, for predicting loot box spending (BF_10_ = 17.35). These analyses add further support to our earlier correlational analyses suggesting that AUDIT scores are not associated with loot box expenditure.

### Exploratory analyses

For further clarity, an exploratory Bayesian linear regression including frequency of alcohol consumption (as the variable with the strongest support for a relationship with loot box spending) and problem gambling symptomatology was conducted on loot box spending. As before, the model of best fit included only problem gambling symptomatology which, when computed as the ratio of evidence for PGSI scores over the interaction, yielded moderate evidence in favour of the problem gambling symptomatology only model (BF_10_ = 5.28) over a model including both main effects and the interaction term. Likewise, there was only anecdotal evidence to suggest a correlation between PGSI and AUDIT scores, with a small effect size (*r*_*s*_ = 0.16, BF_10_ = 1.13). Similar results, albeit less conclusive, were observed when number of standard drinks were used as the independent measures (see analyses in OSF).

Replicating previous research^11,32^ and as seen in Table 2, there was very strong evidence for a small positive relationship between participant PGSI scores and loot box spending. Similarly, there was extreme evidence for a moderate positive relationship between PGSI and RLI scores. There was also extreme evidence of a moderate positive correlation between IGD scores and increased loot box spending. This was also the case for IGD scores and increased risky interactions with loot boxes, which showed a moderate relationship. Evidence for the associations between problem gambling and risky loot box engagement, and excessive gaming and risky loot box use, were essentially definitive.

## Discussion

We investigated the association between alcohol consumption behaviours and loot box spending in a sample from Australia, Aotearoa (NZ) and the US. Contrary to our predictions, our findings suggest that habitually risky and harmful drinking (AUDIT score) had no association with loot box spending, either alone or in concert with problem gambling, or excessive gaming symptomatology. However, concordant with predictions, estimated frequency and number of standard drinks consumed during gaming sessions were positively associated with increased spending and risky interactions with loot boxes. Only a minority of participants (18.59%) engaged with both target behaviours. Critically our measures asked participants to report behaviours over one month and 12-month periods – thus, we are unable to determine the consistency of these behaviours over longer time periods. Further research in this area in representative samples will be needed to determine prevalence on these types of behaviours in the greater gaming population.

Acute drinking, the amount of alcohol consumed during a gaming session, was measured by (a) number of standard drinks consumed during the participant’s most recent gaming session and (b) estimated frequency of concurrent alcohol consumption and gaming. We found a stronger association between acute drinking and risky engagement with, and spending on, loot boxes, than general measures of alcohol consumption behaviour. These findings are largely consistent with current research, suggesting that under the influence of alcohol, individuals who engaged in gambling activities tend to engage in more impulsive and risky spending than sober controls^19^. Exploratory analyses did not reveal evidence for an interaction between problem gambling symptomatology and indices of acute alcohol consumption on loot box spending, suggesting that although the risky behaviours of problem gambling symptomatology, acute alcohol consumption, and loot box spending may co-occur, drinking does not appear to be a causal factor for increased loot box spending.

There appear to be some associations between alcohol consumption and loot box spending, which might be attributed to impaired inhibitory control and impulsivity. However, this association may not be clear-cut, given the stronger association is with risky loot box engagement^18^. These findings, contrasted with the lack of relationship between loot box engagement and AUDIT scores, imply that alcohol alone may not motivate increased spending on loot boxes. This is consistent with previous findings that the impact of alcohol consumption on risk taking is likely nuanced, involving both biological and psychological mechanisms, as well as external motivating factors^22^. Contextual cues may also influence risk taking behaviours, such that gamers may mistakenly believe that loot boxes are not gambling as they occur in an environment that bears less resemblance to traditional gambling venues. These contextual differences may partially explain why the present study observes relationships between loot box engagement and acute drinking. Taken together, the present study provides further evidence for the co-occurrence of addictive behaviours by showing associations between acute alcohol consumption, problem gambling symptomatology, and risky loot box engagement.

### Limitations and future research

Although our data advance our understanding of loot box spending and addictive behaviours, several limitations bear mentioning. First, this study focuses on correlational data and can therefore not determine causality. Further studies are therefore warranted to examine longitudinal data and causal relationships. Second, our measures of standard drinks and frequency of drinks were self-report measures which introduces the possibility of self-report error or other sources of noise into the data. Future research could seek to reduce this in controlled administration of alcohol in lab-based trials. Custom built video games may also be incorporated to manipulate the type and presentation of loot boxes to participants. It is unclear that the psychological conditions of interest – those in which drinking and (risky) loot box purchasing co-occur – could be faithfully recreated in a controlled lab environment. However, it would be possible to test the effects of alcohol intoxication on inhibition for tasks relevant to gaming and in-game spending. Similarly, this study only investigates relationships between alcohol consumption and loot box spending in a western sample. It will therefore be important to conduct cross cultural studies to verify how environmental and cultural factors may affect alcohol consumption and subsequent loot box interactions.

While engaging with loot boxes in video games and alcohol consumption are individually both common activities, to date, little has been investigated about the co-occurrence of these two activities. As a first step toward remedying this gap in the literature, we aimed to identify whether there was a specific subgroup of individuals who engaged with loot boxes and alcohol consumption and whether these behaviours occurred concurrently. We are buoyed that the rate of the target behaviours co-occurring in this sample was low. This is likely because the occurrence of any behaviour (e.g., buying loot boxes) is always, by definition, higher than the frequency of a constellation of behaviours (e.g., drinking and buying loot boxes). As the present study did not employ a representative sample, there is a need for tightly controlled prevalence studies to understand the precise frequency of the co-occurrence of these behaviours. Future studies may also seek to directly target a sample of individuals who consume alcohol while engaging with loot boxes to better understand the potential for the co-occurrence of these behaviours to cause harm (if any), even if the frequency of co-occurrence of the behaviours is low.

Notably, participant scores on the AUDIT (*M* = 4.5, *Mode* = 1) fell well below the cutoff score (8/40) for hazardous and harmful drinking^26^. The low number of individuals with problematic drinking behaviours in the sample could therefore have reduced our ability to detect any association between chronic alcohol use and spending on loot boxes. Thus, it is important to note that this study may be underpowered to sufficiently detect the impact of chronic alcohol consumption on loot box spending. General trends also support findings of reduced rates of alcohol consumption in young people in high income countries, which may have been reflected in this relatively young sample^33,34^.

This study, being the first of its kind, should be replicated and expanded upon to include adolescents in the sample, given the popularity of video games within this age bracket. In particular, it will be important to replicate and rigorously test the exploratory analyses in the present work to ensure the results are robust. Likewise, other potential mediating factors on loot box spending, including impulsivity, warrant further investigation.

## Conclusion

We found evidence to support positive correlations between acute drinking and both riskier engagement with, and increased spending on, loot boxes. This suggests that concurrent alcohol consumption and gaming may be associated with elevated risk factors for gamers of risky engagement with, and/or overspending on, gambling-like mechanisms in video games. We also observed similar elevated risks for those with increased excessive gaming symptomatology. However, results indicated no evidence in favour of correlations between more chronic alcohol consumption habits of participants and riskier engagement with loot boxes, though it is possible that the low levels of chronic drinkers in the present study may have reduced our ability to detect such associations. Further research investigating the association between drinking behaviour and loot box engagement is warranted to establish the exact nature of these relationships.

## Data Availability

Data available via OSF: https://osf.io/fwp4t/files/osfstorage/6758d7742158a00950f73a53.
